# Midwakh-Associated Acute Lung Injury (MALI) in a 14-Year-Old Male: A Case Report

**DOI:** 10.7759/cureus.31644

**Published:** 2022-11-18

**Authors:** Angel Varughese, Vijay C Vinod, Suneel Kumar Pooboni, Rania Abusamra

**Affiliations:** 1 Paediatric Intensive Care, Mediclinic City Hospital, Dubai, ARE; 2 Medicine, Queen's Hospital, London, GBR; 3 Paediatric Pulmonology, Mediclinic City Hospital, Dubai, ARE

**Keywords:** alternative tobacco products, interstitial pneumonitis, midwakh, smoking, paediatric, ards, acute lung injury

## Abstract

Midwakh is one of the most used alternative tobacco products (ATPs) in the UAE, particularly among adolescents. We report a 14-year-old adolescent male, who presented with acute lung injury within 24 hours following the first attempt at Midwakh smoking. A high-resolution computerized tomogram of the chest (HRCT) showed bilateral interstitial pneumonia and patchy consolidation. Flexible bronchoscopy revealed bilateral petechial haemorrhages with oedematous bronchial walls. Mechanical ventilation was required for two weeks. The criteria for moderate acute respiratory distress syndrome (ARDS) were met, and a good response was achieved to a high dose of steroids, and ultra-protective mechanical ventilation with the prone position. Significant clinical and radiological recovery was achieved at three months. As per the literature reviewed, this is the first case of Midwakh-associated acute lung injury reported to date. We emphasize that physicians should be well informed about the use of ATPs and their potential severe complications.

## Introduction

As per the World Health Organization (WHO) Global Youth and Adult Tobacco Survey, there is an alarming increase and growing concern about alternative tobacco products (ATPs) [1]. Common ATPs range from electronic cigarettes, hookah or shisha, midwakh, and various smokeless tobacco products. Midwakh and dokha are two terms that are often used interchangeably. Smoking midwakh is increasing in the Gulf and particularly in the United Arab Emirates (UAE) [2], among adolescents and school students. It is the most used ATP in UAE, next only to cigarettes in overall tobacco use [3].

Smoking tobacco exposes the lungs to severe detrimental effects on epithelial and endothelial function [4], in keeping with acute lung injury (ALI) [5] and leading to acute respiratory distress syndrome (ARDS). This was encountered in 2019-2020 when over 2800 eCigarettes or vaping-associated lung injury (EVALI) cases of hospitalization or deaths were reported to the Centre for Disease Control (CDC) from all 50 states in the United States of America (USA), with ages ranging from 13-85 years [6].

We report a 14-year-old adolescent male, who presented with acute lung injury and ARDS following a single session of midwakh smoking, a presentation very similar to EVALI. To our knowledge, this is the first case of midwakh-associated acute lung injury (MALI) ever reported to date.

This article was previously posted to the Authorea preprint server on September 05, 2020.

## Case presentation

**Figure 1:**
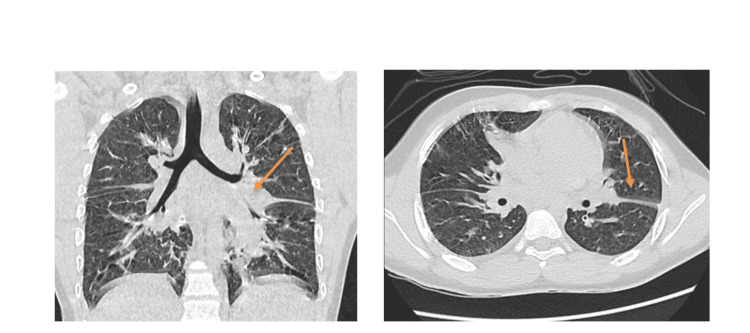
High-Resolution CT (HRCT) coronal and axial views showing bilateral ground glass appearance with septal thickening; patchy consolidation in the right middle lobe and in both lower lobes

We received a young, previously apparently healthy 14-year-old boy, who presented to his local hospital in 2020 with rapidly developing breathlessness, cough, and fever. Symptoms started 24 hours post-smoking midwakh, mixed with crushed Damas tree leaves (Conocarpus lancifolius). He had no known allergies, and there was no history of travel. Informed consent was obtained from the patient and parents for this case report. On admission, he underwent a series of investigations including a COVID-19 polymerase chain reaction (PCR) test; started on empirical broad-spectrum antibiotics and oseltamivir and underwent a high-resolution computerized tomogram (HRCT) of the chest, which demonstrated interstitial pneumonia with patchy consolidation and ground-glass appearance (Figure 1). Within 24 hours, he required intubation and mechanical ventilation due to respiratory failure with significant hypoxia.

The patient was transferred to our centre on day four with hypoxemia (PaO_2_ 77.9 mmHg) despite being on volume support-assist control ventilation with positive-end-expiratory pressure (PEEP) of 8 cmH_2_O, tidal volume (TV) of 400 ml (7 ml/kg) and 70% fraction of inspired oxygen (FiO2). Auscultation of the chest revealed bilateral decreased air entry and crackles.

Flexible bronchoscopy was performed on day five, revealing bilateral erythema, scattered petechial haemorrhages, and oedematous bronchial walls besides a mild increase in airway secretions. Broncho alveolar lavage (BAL) microbiology culture and viral PCR, including the COVID-19 PCR, were negative. BAL cell count differentiation had 71% neutrophils, 5% lymphocytes, 6% monocytes, 18% macrophages, and no eosinophils. No smoking dust, tar or nicotine was seen in gross bronchoscopy or in BAL.

**Figure 2:**
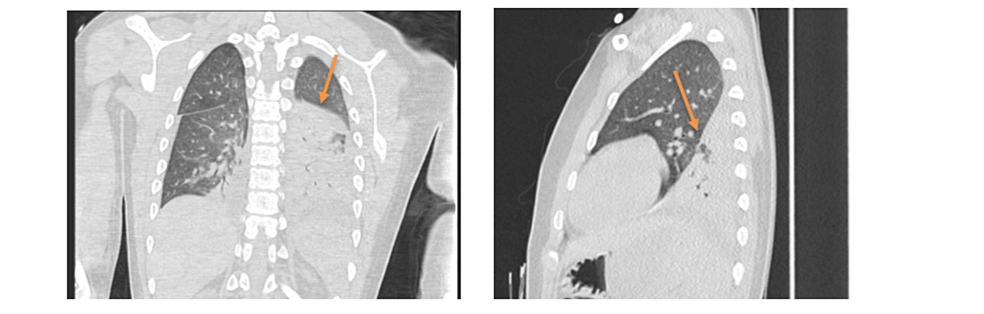
High-resolution computed tomography (HRCT) coronal and sagittal views on day 10 of hospital admission Both lungs show diffuse ground-glass density. Collapse-consolidation is seen in the right posterior basal segment and left lower lobe with an air-bronchogram. Small effusions are present bilaterally.

The above finding indicated interstitial pneumonitis with moderate ARDS (bilateral infiltrates on radiographs, PaO_2_/FiO_2_ ratio <200 mmHg on PEEP 8 and no cardiogenic cause). He was managed with ultra-protective mechanical ventilation (TV ~5 ml/kg) in addition to neuromuscular blockade and sedation to support mechanical ventilation. Continuous infusion of low-dose furosemide was commenced for a positive cumulative fluid balance of four litres. The patient was commenced on a five-day course of the following anti-inflammatory medications: intravenous methylprednisolone one gram/day (15 mg/kg/day), oral hydroxychloroquine (300 mg per day) and oral azithromycin (500 mg per day). During the paediatric intensive care unit (PICU) admission course, he developed hypertension (probably due to intravenous corticosteroids) and pre-renal failure for which anti-hypertensive medications were administered, and diuretics were discontinued. The patient was rehydrated following this episode, balancing the perfusion of his kidneys and lungs. Antibiotics were de-escalated because of negative microbiology results. On day 10, high-peak inspiratory pressure ventilation (PIP 35 cmH_2_O) was still required despite the high dosage of methylprednisolone. A chest HRCT scan (Figure 2) was repeated, showing left lower lobe consolidation, right lower lobe collapse and interstitial lung changes with ground glass appearances.

Subsequently, a prolonged period of prone ventilation was initiated in addition to regular chest physiotherapy. Eventually, there was a significant improvement in the ventilator requirements until extubation on day 14. Spirometry one week post-extubation revealed a restrictive pattern with forced vital capacity (FVC), forced expiratory volume in one second (FEV1), and FEV1/FVC being 2.15 L (46.8%), 2.02 L (53.3%) and 94.09 % (112.8 %), respectively. A mild global weakness, features of critical illness myopathy and peripheral neuropathy were noted. The patient was discharged on a tapering dose of prednisolone.

Follow-up at three months post-discharge showed resolving respiratory symptoms, regained effort tolerance, normalizing a chest HRCT (Figures 5, 6) and significantly improved spirometry with FVC of 3.8 L (91% predicted), FEV1 of 3.1 L (89% predicted), diffusion capacity (DLCO) of 6.3 L (70% predicted) and normal 6 min walk test (heart rate of 110 bpm increasing slightly to 121 bpm post the walk, and oxygen saturation (SpO_2_) of 98% unchanged post the walk test).

**Figure 3:**
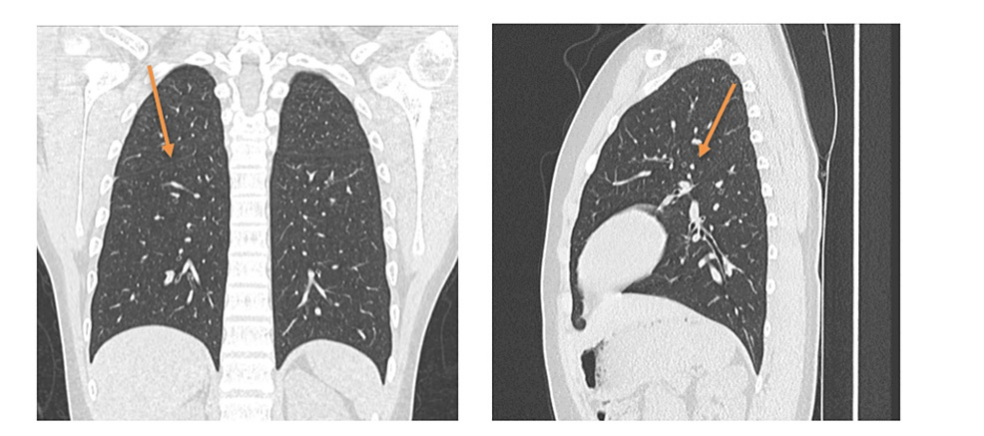
High-resolution computed tomography (HRCT) coronal and sagittal views done three months post-discharge from the hospital show full recovery of the lungs

## Discussion

**Figure 4:**
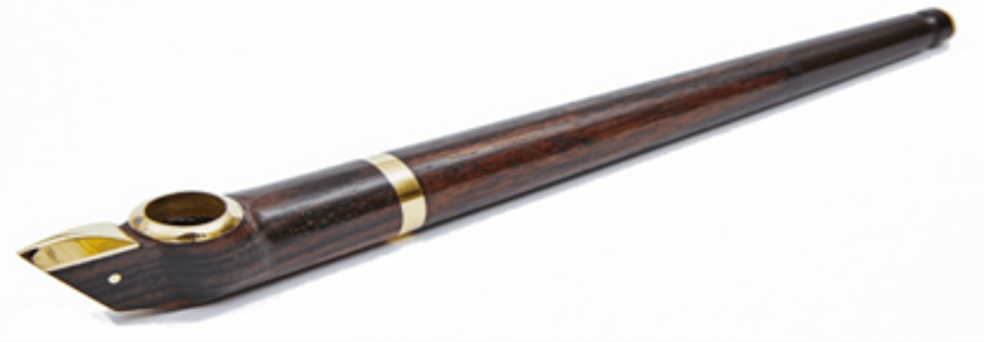
Midwakh pipe

We present a 14-year-old patient with ARDS (the most severe form of ALI), due to smoking midwakh. Midwakh is manufactured by drying tobacco leaves and mixing them with herbs and spices to enhance the flavour. It is smoked through a small pipe, which has three parts, a mouthpiece, a stem and a bowl (Figure 4). The bowl can contain approximately 0.5 grams of dry tobacco, enough to finish in two inhalations [7]; therefore, an individual ends up smoking midwakh 20-25 times a session on average [8].

Nicotine levels in midwakh tobacco products were 170.76%, 218.07%, 128.57% and 193.33% higher in comparison with cigarettes, chewing tobacco, snuff tobacco and electronic cigarettes, respectively, and its tar content is the highest when compared to shisha and cigarettes [9]. This may explain the exaggerated lung injury in our case post the first attempt of smoking midwakh. In addition, midwakh tobacco products contain harmful levels of cobalt, chromium, cadmium, iron, lead, carcinogens and central nervous system (CNS) depressants [10]. Traditional midwakh pipes contain no filters; hence, these toxicants can quickly enter the lungs. Midwakh smokers may add herbs, spices, dried fruits and leaves of native plants (like the Damas tree leaves) to tobacco.

Toxic inhalational lung injury may result in a spectrum of respiratory disorders like bronchopneumonia, pulmonary oedema, interstitial lung disease, reactive airways dysfunction syndrome, obstructive airway disease and bronchiolitis obliterans organizing pneumonia (BOOP) [11]. Fatal cases following significant exposure were associated with laryngeal oedema, airway obstruction, non-cardiogenic pulmonary oedema/ARDS and secondary infection in the setting of the diffuse airway and pulmonary parenchymal damage [11]. Also, the resolution of initial acute pneumonia can be followed by the gradual onset of airway obstruction, which has to be watched during the follow-up.

During 2019-2020, there were several reports of clusters of patients presenting with e-cigarette/vaping‐associated lung injury (EVALI) outbreak in the USA. The clinical and radiological findings in our case were in keeping with the reported EVALI. Patients with EVALI demonstrated a heterogeneous collection of pneumonitis patterns that included acute eosinophilic pneumonia, organizing pneumonia, lipoid pneumonia, diffuse alveolar damage and ARDS, diffuse alveolar haemorrhage, hypersensitivity pneumonitis, peribronchiolar granulomatous pneumonitis and the rare giant-cell interstitial pneumonitis [12]. Symptoms like cough, shortness of breath and fever can rapidly worsen within 24-48 hours of initial presentation, as happened in our case.

These patients were treated with systemic steroids to reduce the inflammatory response. Steroids showed an improvement in the clinical condition in almost 82% of treated patients [13]. Some patients have relapsed during corticosteroid tapering, and some have had persistent hypoxemia (SpO2 <95%) requiring home oxygen at discharge [13]. Our patient responded well to steroids and no relapse occurred during dose tapering. Hydroxychloroquine (HCQ) was given for five days off-label for evolving interstitial changes on presenting chest HRCT and for suspecting COVID-19 infection initially with the available evidence at that time of its role in treating ARDS due to COVID-19.

ARDS has an incidence of 30-80 cases per 100,000 populations [14]. As per the 2012 Berlin definition, it is divided into three categories according to the severity of hypoxemia as the mild (PaO_2_ /FiO_2_ 200-300 mmHg), moderate (PaO_2_/FiO_2_ 100-200 mmHg) and severe (PaO_2_/FiO_2_ < 100 mmHg) forms of ARDS.

Despite a much-improved understanding of ARDS pathophysiology, the efficacy of used therapeutic approaches is limited. In our case, although ultra-protective lung ventilation (TV 4 to 5 ml/kg) was used to avoid barotrauma, his PIP was reaching up and beyond 35 cm H_2_O. To improve ventilation in our case we resorted to prone positioning and the use of neuromuscular blocking agents. Prone positioning was useful in our case by enabling better ventilation/perfusion matching, more homogenous distribution of ventilation and the recruitment of dorsal regions. The prone position also reduces ventilator-induced lung injury [15].

The use of neuromuscular blocking agents improves patient-ventilator synchrony and reduces oxygen consumption leading to improved survival [16]. However, when given alone with steroids, it can exacerbate weakness due to critical illness myopathy and polyneuropathy [17]. In our case, it could not be avoided, as we had to balance the risk of Intensive care unit-acquired weakness with the potential benefit of neuromuscular blockers and systemic glucocorticoids in the severely lung-injured patient.

An interesting study highlighted various new outcomes that need to be studied in pediatric acute respiratory distress syndrome (PARDS) like long-term pulmonary function, risk of pulmonary hypertension, nutrition status and growth, PICU-acquired weakness, neurocognitive development, functional status and health-related quality of life (HRQOL) [18]. A cohort study conducted on 316 mechanically ventilated paediatric patients concluded that 23% developed new morbidities due to residual organ dysfunction, treatment complications, etc [19].

There have been numerous studies on long-term outcomes in adult ARDS. One study with potential application to paediatrics illustrated that half of the patients had persistent functional disability 12 months after discharge [20]. The 6-minute walk test (6MWT) helps assess global physical function, i.e., lung and cardiac function and muscle strength. The mean distance walked in the 6MWT increased significantly over the first year when followed up at three months and 9-12 months after discharge.

Our patient had significantly reduced spirometry parameters upon discharge from the PICU, which got almost normalized within three months post-discharge from the hospital.

## Conclusions

We report the first-ever case of midwakh-associated acute lung injury (MALI), which posed a diagnostic challenge during the COVID-19 pandemic period. It was a diagnosis of exclusion and was based on a history of clinical presentation, signs and symptoms and radiological features. It was a close mimic of the EVALI epidemic in the USA. Our case responded to a high dose of steroids, prone ventilation with ultra-short lung-protective settings. Significant recovery was achieved at three months though the long-term effects must be watched for.

As of 2022, there are no such clinical studies on Midwakh to identify the long-term risks attributable to it. We recommend additional research and studies to identify the pathophysiology of acute lung injury and the acute and long-term effects of midwakh smoking. We request physicians and paediatricians to work closely with adolescents and their parents to discourage tobacco products. Appropriate public health education and strictly obeying the country’s tobacco control laws can prevent a MALI epidemic in the region and protect the younger generation.
